# “If you understand you cope better with it”: the role of education in building palliative care capacity in four First Nations communities in Canada

**DOI:** 10.1186/s12889-019-6983-y

**Published:** 2019-06-17

**Authors:** Holly Prince, Shevaun Nadin, Maxine Crow, Luanne Maki, Lori Monture, Jeroline Smith, Mary Lou Kelley

**Affiliations:** 1 Centre for Education and Research on Aging & Health, Lakehead University, 955 Oliver Rd, Thunder Bay, Ontario P7B 5E1 Canada; 2 Centre for Rural and Northern Health Research, Lakehead University, 955 Oliver Rd, Thunder Bay, Ontario P7B 5E1 Canada; 3 Department of Health Sciences, Lakehead University, 955 Oliver Rd, Thunder Bay, Ontario P7B 5E1 Canada; 4 Netaawgonebiik Health Services, Naotkamegwanning First Nation, ON Canada; 5 Fort William First Nations Health Centre, Fort William First Nation, ON Canada; 6 Six Nations Long Term Care/Home and Community Care Program, Six Nations of the Grand River Territory, ON Canada; 7 Peguis Home and Community Care, Peguis First Nation, MB Canada; 8 School of Social Work, Lakehead University, 955 Oliver Rd, Thunder Bay, Ontario P7B 5E1 Canada

**Keywords:** First Nations, Aboriginal, Indigenous, Palliative care, Education, Community development, Capacity development, Public health approach

## Abstract

**Background:**

In Canada, there is a growing need to develop community-based, culturally appropriate palliative care for Indigenous people living in First Nations communities. The public health approach to palliative care, which emphasizes community-based initiatives, is especially relevant in First Nations communities because care is grounded in their distinct social and cultural context. Central to the public health approach are educational strategies that strengthen communities’ capacity to care for their vulnerable members as they die. This paper presents community-based research conducted with First Nations communities in Canada that aimed to assess and address local palliative care educational needs to improve community capacity in palliative care.

**Methods:**

Participatory action research (PAR) was conducted with four First Nations communities in Canada over a six-year period (2010–2016). The research occurred in three phases. *Phase 1:* focus groups, interviews and surveys were employed to assess community specific needs and resources. *Phase 2:* recommendations were developed to guide the PAR process. *Phase 3: e*ducational resources were created to address the identified educational needs. These resources were implemented incrementally over 4 years. Ongoing process evaluation was employed, and revisions were made as required.

**Results:**

Educational needs were identified for patients, families, community members and internal and external health care providers. A wide and comprehensive range of educational resources were created to address those needs. Those culturally appropriate educational resources are available in a very accessible and useable workbook format and are available for use by other Indigenous people and communities.

**Conclusions:**

This research provides an example of the public health approach and offers implementation strategies around palliative care education. This paper contributes to the international literature on the public health approach to palliative care by presenting a case study from Canada that includes: conducting a culturally appropriate assessment of educational needs, creating recommendations, facilitating development and implementation of educational resources in the community to improve community capacity in palliative care.

**Electronic supplementary material:**

The online version of this article (10.1186/s12889-019-6983-y) contains supplementary material, which is available to authorized users.

## Background

Providing access to quality palliative care is increasingly being recognized as an international public health issue, including for people living and dying in First Nations communities in Canada. The public health approach to palliative care, which emphasizes community level initiatives, is especially relevant for people living in First Nations communities because care is grounded in their distinct social and cultural context. Central to the public health approach are educational strategies that strengthen communities’ capacity to care for their vulnerable members as they die.

The Improving End-of-Life Care in First Nations Communities (EOLFN) was a participatory action research project conducted in Canada to build community capacity to provide palliative care in four First Nations communities [1, 2]. This paper focuses on the project data related to palliative care education, and describes the community-led educational initiatives facilitated to address educational needs during the EOLFN research.

The paper begins by situating palliative care within a public health paradigm. Next, the research context is described which includes the need to develop palliative care in First Nations communities, and an overview of the EOLFN project during which the education data presented in this paper were collected. The details of the research pertaining to community educational strategies are presented next. The paper concludes with a discussion of the implications of this palliative care education research for research, policy, and practice.

### The public health approach to palliative care (health promoting palliative care)

Palliative care is an approach to health care aimed at improving the quality of life of people living with a life-threatening illness and their families [3]. Traditionally, palliative care was limited to care provided by medical experts in institutional settings when death was imminent [4]. Today palliative care is viewed as an approach that is an enhancement to primary care. It extends far beyond specialized end-of-life care and is applicable much earlier in the illness trajectory [4–7]. To adapt to an aging population and the increased numbers of people living with and dying from chronic disease and frailty, there has been an international shift towards a de-medicalized, population-based public health paradigm which normalizes death and expands the responsibility for palliative care from institutions to communities [8–14]. Culture plays a key role in the palliative approach to care since the approach is grounded in the social practices and beliefs of the community [15].

The public health approach (known as health promoting palliative care) promotes “health in the face of death” [12] and emphasizes the role of community in the provision of palliative care [12, 16–18]. This approach acknowledges that most people do not require institutionalized care at the end of life, and instead die in their communities supported by family, friends, neighbours and non-specialist health care providers [19]. As illustrated in Abel and colleagues’ Circles of Care framework [20], the public health approach situates the person with a life-limiting illness at the centre of inner and outer networks of friends, family and neighbours who provide direct and indirect care and support; surrounded by community, service provision organizations and policy which support community care. This approach can be seen internationally in the ‘compassionate communities’ movement which advocates that while necessary, health services alone are not sufficient to provide quality care for people who are dying [9, 21]. There is growing evidence of the positive outcomes of health promoting palliative care [18].

Successful implementation of the public health approach requires appropriate policies, adequate drug availability, implementation of palliative care services across multiple levels, and education [22]. Central to this approach are strategies aimed at strengthening communities’ capacity to care for people who are dying [9]; the emphasis is on supporting communities to care for their people at the end of life [20]. Relevant to this capacity building, recent research has emphasized the need for family and community education to decrease anxiety, and increase the sense of self-efficacy, mastery and preparedness amongst family and community [23]. Building family and community caregiving capacity is particularly important to First Nations communities where formalized palliative care services are lacking.

### Social context: the need to develop palliative care in First Nations communities

Canada’s Indigenous peoples include three groups: First Nations, Inuit, and Metis people. First Nations represents the largest group of Indigenous people in Canada. Many (approximately 474,000) First Nations people live in a First Nations community [24]. There are 618 First Nations communities in Canada, many of which are small and located in rural and remote regions [25]. First Nations communities are highly diverse in language, culture, infrastructure and services, and partnerships with non-Indigenous services outside of the community. Thus, community capacity development initiatives need to be community-based, community-driven and customized to local context.

The need for quality palliative care services for First Nations peoples is urgent due to an aging population and a high burden of chronic and terminal disease [26, 27]. People living in First Nations communities do not want to seek palliative care services outside of their community; they want the opportunity to die in the communities where they have lived out their lives [28–31]. However, due to jurisdictional issues related to health service delivery [32, 33], people in First Nations communities lack the health services and resources to meet the needs of their community members who are very sick. Consequently, First Nations people with complex and high intensity needs must leave their communities to seek care, and most often die in hospitals or long-term care homes outside of their community, separated from family, community, and culture [34–36].

First Nations people have long-standing cultural practices surrounding the end of life. Death is seen as sacred and a natural part of the life cycle, and care and support are provided by family and community [37–40]. There are long-standing traditions for preparing for death and established social processes for supporting community members through dying, loss, grief and bereavement [29, 31–33]. Connection to the land (the home land or home community) is especially important for people who are dying [31, 40].

Unfortunately, the process of colonization in Canada relocated First Nations people and dispersed extended families. It forced First Nations people to be disconnected from their land, culture, language and community. Many First Nations children (ages 4–16 years) were forcibly removed from home and placed into government funded Christian residential schools (1870–1996). Many other children were removed from their families and communities by the child welfare system and placed in non-Indigenous foster and adoptive homes. Over one hundred years of colonization thus disrupted natural helping networks and interfered with intergenerational transmission of cultural knowledge and community caregiving traditions [41]. It further created distrust of Canadian social institutions, including the health care system [42]. Now, many First Nations communities in Canada are reclaiming their cultural knowledge about caregiving and end of life issues, and seeking new knowledge of palliative care that would enable them to care for their Elders and loved ones at home until the end of life [28, 30, 38].

The health promoting palliative care approach is especially relevant to developing palliative care in First Nations communities because it moves away from the medicalized approach of industrialized nations, often known as Western medicine. The focus is on holistic community care for people with life-limiting illnesses that is provided by family, friends, and non-specialist health care providers in the community (as opposed to hospitals or other institutions). Successful implementation of the public health approach requires community capacity building and education, as described in this research.

### Research context: the improving end-of-life care in First Nations communities project

The research presented here was conducted as part of the “Improving End-of-Life Care in First Nations Communities (EOLFN)” project conducted in partnership with four First Nations communities in Canada [1]. The goal of that research was to improve end-of-life care through developing palliative care programs and teams in each of the partner communities to support community members who are seriously ill with a life-threatening condition. Over a six-year period (2010–2016), the EOLFN researchers worked with the partner communities to implement a community development process and build palliative care capacity.

All four communities created palliative care programs uniquely designed to their culture and context. Policy recommendations were created for health care decision makers. Research outcomes can be found in publications [1, 43–46], community reports [47–50], and a workbook for other First Nations communities entitled “Developing Palliative Care Programs in First Nations Communities” [51]. This paper by the EOLFN researchers adds new data on education to the existing dissemination.

**Fig. 1:**
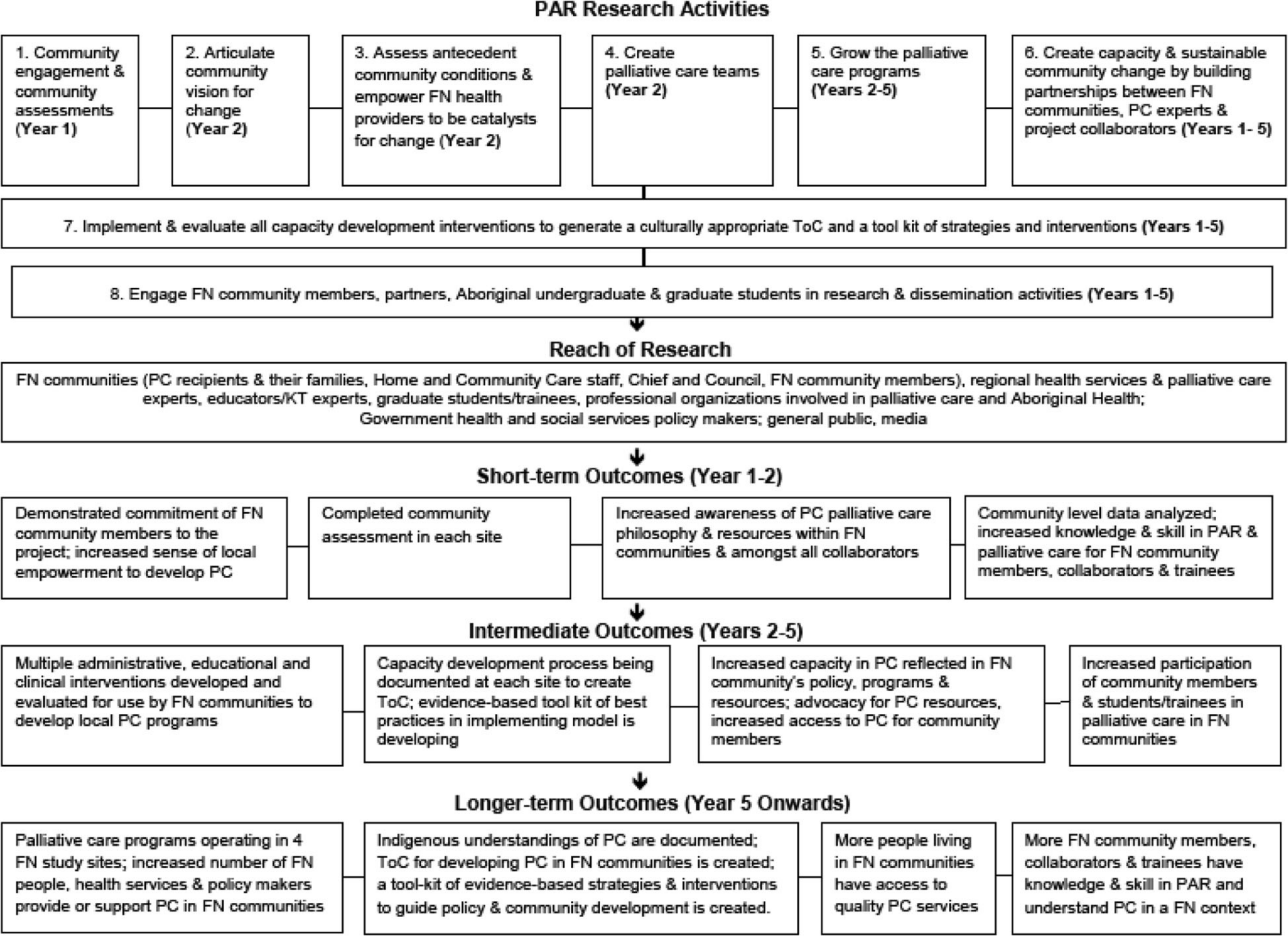
Improving End-of-Life Care in First Nations Communities (EOLFN) Project Logic Model

An understanding of the overall EOLFN project and research process is relevant to this paper. Thus, before presenting the education data that is the focus of this paper, important context is provided to the reader through an overview of the EOLFN communities and research process. In addition, the EOLFN project logic model is provided in Fig. 1.

#### Community descriptions

**Fig. 2:**
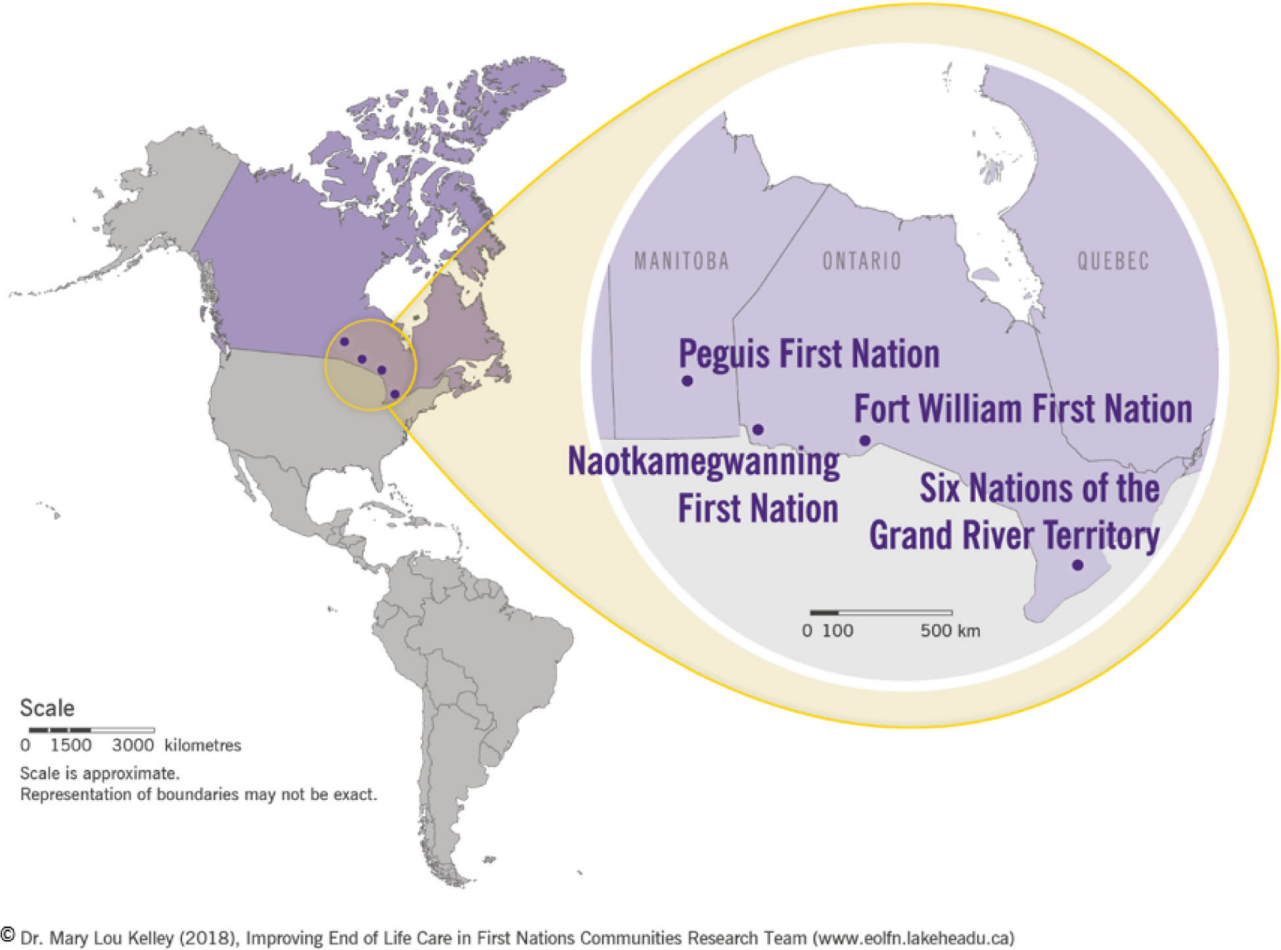
Geographical Location of the Four First Nations Partner Communities

The four communities were chosen as study sites for maximum variation which strengthens study conclusions. Communities were in different regions of Ontario (*n* = 3) and Manitoba (*n* = 1), each having different provincial health services (Fig. 2).

The community demographics varied in population size, proximity to an urban health service center, level of community infrastructure, and cultural identity (Table 1). Retention of traditional language within the communities, an indicator of maintaining cultural tradition, ranged from retention rates over 50% to communities currently undertaking language revitalization (e.g. language immersion programs in the schools to promote and preserve their traditional languages). To be included in the research, community leaders also needed to be committed to building capacity for palliative care in their community and consent to partner with the research team to undertake the research for 6 years.

**Table 1:** Community Demographic Characteristics, 2008^a^

Community Attribute	Naotkamegwanning First Nation (NFN)	Fort William First Nation (FWFN)	Peguis First Nation (PFN)	Six Nations of the Grand River(SNGRT)
Population^b^	675	878	3558	11,634
Proximity to an urban centre with hospital/specialized health services (urban centre)	100 km (Kenora) 537 km (Thunder Bay)	2 km (Thunder Bay)	190 km (Winnipeg)	25 km (Hamilton) 19.3 km (Brantford)
Level of local health infrastructure	- Minimal & need to travel 1 h for services - New Elders residence - Health Centre - Home and Community Care Program^c^ - Long term care program^d^	- Good & easy access to hospital & services in Thunder Bay - Services mainly external - Home and Community Care Program (Dilico)^c^ -Long term care program^d^	- Good & easy access to hospital in Hodgson (16 km - Hub for smaller First Nations	- Excellent & easy access to hospitals and hospices in Hamilton and Brantford - Home and Community Care Program - Long term care program^d^ - Long term care facility - Assisted Living Residence - Numerous other programs and services available in the community
Cultural Identity	Ojibway	Ojibway	Ojibway and Cree	Haudenosaunee

^a^These were the community attributes at the time the needs assessments were being planned and conducted ^b^ Population is defined as the number of people living in the community; total populations (i.e., total number of community members, including those who reside outside of the First Nations community) were: Naotkamegwanning, 1142; Fort William, 1854; Peguis, 8558, Six Nations, 23,289

^c^First Nations Inuit Health Branch, Home and Community Care Program (home care includes nursing, personal support workers)

^d^Long term care program (includes home makers and respite care)

#### Research process

Prince and Kelley’s Integrative Framework for conducting palliative care research in First Nations communities [52] summarizes the research approach used in the EOLFN research. This framework integrates five well-established research components that, used together, respect the unique needs and culture of First Nations communities. The integrated components are community capacity development, cultural competence and safety, participatory action research, partnerships and ethics (including adherence to the principles of Ownership, Control, Access and Possession which are sanctioned by the First Nations Information Governance Centre to ensure self-determination in research concerning First Nations [53]).

The research paradigm adopted for the EOLFN project was participatory action research (PAR). PAR generates practical and theoretical knowledge by implementing a social change process that benefits participants [54]. This paradigm differs from conventional research paradigms in: i) its understanding and use of knowledge; ii) its relationship with research participants; and iii) the introduction of change into the research process [55, 56]. Knowledge is co-created by the researchers and participants, and participants work *with* researchers to determine the methods and techniques most relevant [56]. Consistent with the PAR approach, all aspects of the research and palliative care program development were controlled by community members.

**Fig. 3:**
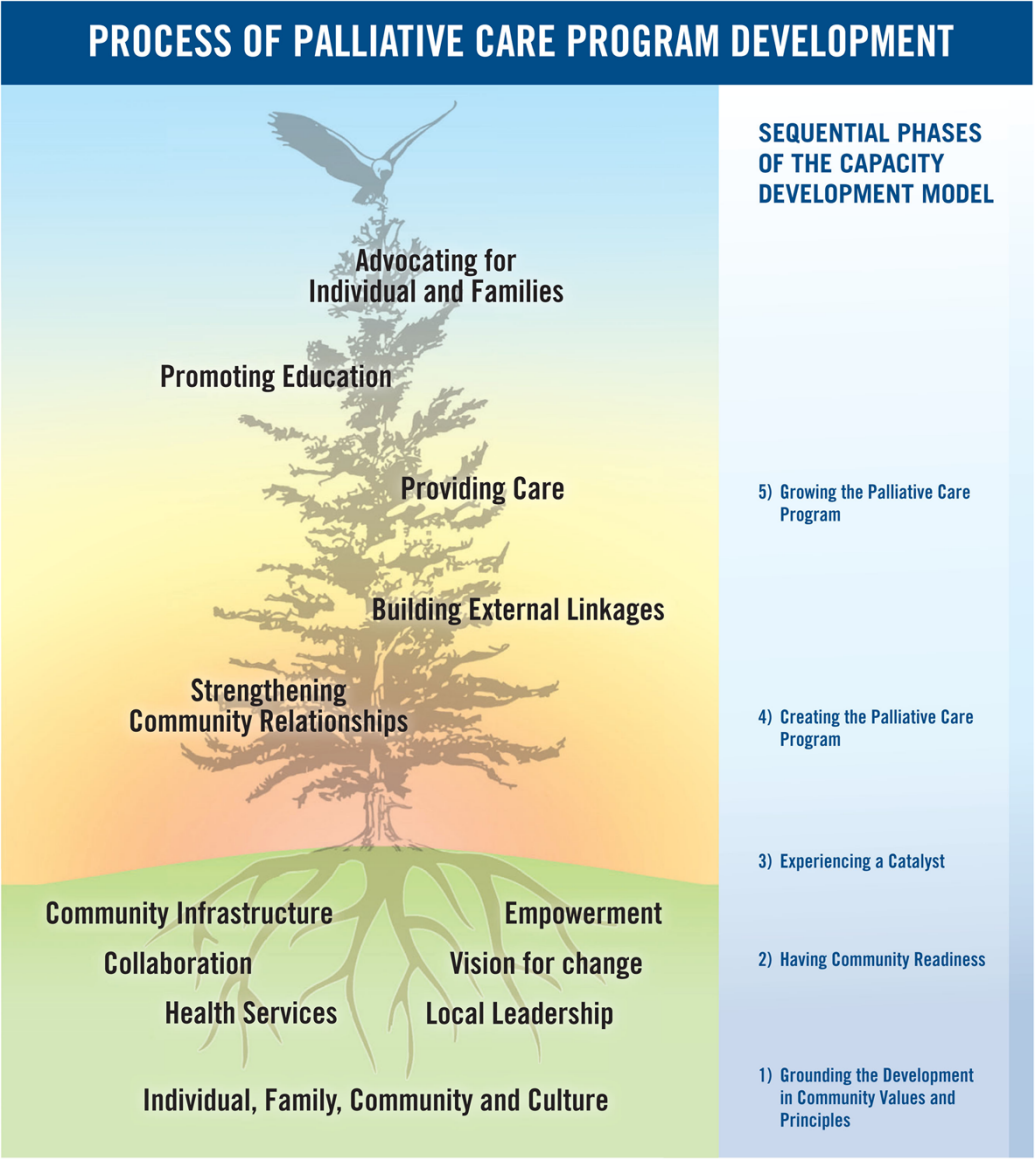
Process of Palliative Care Development in First Nations Communities. Kelley’s community capacity development model [57] adapted to the First Nations culture and context [1]

The First Nations participants adapted Kelley’s model for rural community capacity development in palliative care [57] to be culturally appropriate [1]. The key principles of the model are: change is incremental and dynamic; change takes time; development builds on existing resources and is essentially about developing people; development needs to be ‘bottom-up’, not imposed from outside; and development is ongoing (57). Building on these principles, the First Nations model outlines five phases of community palliative care development: i) grounding the program in community values and principles, (ii) having community readiness, (iii) experiencing a catalyst, (iv) creating a palliative care program, and (v) growing the palliative care program (Fig. 3).

The phases represent a gradual transformative process. Each phase has several tasks that must be accomplished, culminating in the delivery of a palliative care program that is mobilized through strengthening community relationships, building external linkages with palliative care experts and resources, providing local palliative care; advocating for individuals and families and promoting education. Education is a key issue in this capacity development model and is the focus of this paper.

### The present paper: community educational strategies of the EOLFN project

The purpose of this paper is to report on the process and outcomes of the educational components of the EOLFN research. Specifically, this paper presents the process of assessing the palliative care education needs, and the implementation of resources to address the identified educational needs in the four participating communities. This paper contributes to the international literature on the public health approach to palliative care by offering a case study from Canada that includes: conducting a culturally appropriate assessment of educational needs, creating recommendations, facilitating development and implementation of educational resources in the community to improve community capacity in palliative care.

## Methods

The process of assessing and addressing the community palliative care education needs in each of the participating communities unfolded in three phases: i) assessing educational needs in each community; ii) developing recommendations based on the findings, and iii) developing and implementing educational resources to address the identified needs. The details of each phase are described next.

### Phase 1: assessing educational needs

Following initial engagement work that created a community advisory committee of local Elders and Knowledge Carriers, leaders and health care providers in each community, a community assessment was conducted in each community to help inform the local capacity development process. The assessments were comprehensive and assessed the following domains: understandings of and experience in providing palliative care; barriers and supports in the community; current palliative care practices and available resources; and (the focus of this paper) the educational needs of formal and informal community caregivers.

The community assessments were conducted between January 2011 and September 2012. A mix of quantitative and qualitative data were collected sequentially, beginning with surveys, followed by focus groups and interviews in each community. Consistent with the PAR approach and to ensure cultural appropriateness, all instruments were approved by the community advisory committees. Data were collected by a First Nations community facilitator chosen by the advisory committee and trained by the researchers. Translation was provided as needed by a member of the local advisory committee. These First Nations community leaders are competent in the local language and are trusted by community members to report accurately.

#### Surveys

A purposive sample of key informants in each community was invited to participate in a survey that was designed to understand and assess the community’s readiness to develop and access a local palliative care program. Key informants included Elders and Knowledge Carriers, community members, members of Band Council and internal health care providers (a community member who is also a health care provider within the community). Informants were chosen by the community advisory committee.

This original survey was developed by Prince and Kelley and has been evaluated and modified over 10 years working with First Nations communities. The survey items included basic demographics and those issues that previous research has demonstrated will influence people’s access palliative care services: respondents’ opinions, experiences, values and beliefs, and the availability of palliative care education and availability of health services.

Although the survey contained a mix of closed and open-ended questions, most questions were closed-ended. Earlier versions of the survey contained mostly open-ended (exploratory) questions; however, palliative care is a medical concept unfamiliar to First Nations people, and there are not comparable ideas or words for many palliative care concepts in First Nations’ languages. Therefore, the survey was adapted to a more structured format which was found to be more effective for assessing community understandings and needs related to palliative care. The survey employed in this research has now been used successfully in approximately 25 First Nations communities in Canada. The survey can be found in the Additional file 1, and is available on the EOLFN project website [2].

There were 94 surveys completed across the four communities. Respondents ranged in age from under 30 to over 60 years; most were female (See Table 2). Analysis of the survey data included the calculation of descriptive statistics on the numerical data obtained from the closed-ended survey questions, and thematic analysis of the qualitative data from the open-ended question.

**Table 2:** Demographic Characteristics of Key Informant Survey Participants, by Community

Community	# of Respondents	Demographics						
		Gender		Age				
		Female	Male	> 30 yrs	31–40 yrs	41–50 yrs	51–60 yrs	< 60 yrs
FWFN	24	21	3	2	3	7	3	9
NFN	21	14	7	3	2	3	3	9
PFN	29	22	7	0	3	10	10	6
SNGRT	20	13	7	0	3	1	6	10
Total	94							

*FWFN* Fort William First Nation, *NFN* Naotkamegwanning First Nation, *PFN* Peguis First Nation, *SNGRT* Six Nations of the Grand River Territory

#### Focus groups & interviews

Following analysis of the survey data, individual interviews and focus groups were conducted in each community to elaborate on the survey findings. Focus groups were the primary method; however individual interviews were conducted with key informants who could not attend the scheduled focus groups or whose preference was to conduct the interview one-on-one. External health care provider interviews were also conducted individually. Purposive sampling, directed by the community advisory committee, was used to recruit participants and four sample groups were targeted: community members, Elders and Knowledge Carriers, internal health care providers, and external health care providers (See Table 3).

**Table 3:** Interview and Focus Group Sample Groups

Sample Group	Definition
Community Member	A member of one of the participating First Nations community
Elder/Knowledge Carrier	A member of the community having status as being knowledgeable either due to age or immersion into the traditional cultural practices of the community
Internal Health Care Provider	Community member who is also a health care services provider within the First Nations community, or a health care provider who comes regularly to the community, has a long-standing relationship and is trusted by the community
External Heath Care Provider	Non-community members (primarily non-Indigenous) who provide health care services either in or outside of the community to one of the 4 First Nations community partners

The focus groups and interviews were guided by semi-structured interview guides with questions that were designed to complement and probe participants for a more in-depth response to the questions posed in the surveys (See Additional file 1). To facilitate discussion, the focus groups were preceded by a presentation of the survey findings – this was helpful in providing context and allowed participants to react to the survey findings. The focus groups and interviews were audio-recorded. The audio recordings were manually transcribed verbatim, including using interpreters when First Nations languages were spoken.

A total of 62 focus groups and interviews were conducted across the four communities with a total of 185 participants. Table 4 outlines the number of focus groups, interviews and participants (by sample group) for each community. To decrease burden and increase the cultural safety of the research process, and because it was not essential to the research purpose, no further demographic data were collected from focus group and interview participants.

**Table 4:** Number of Participants in Focus Groups and Interviews by Sample Group, by Community

Community	# of Interviews/Focus Groups Conducted	# of Participants			
		Community Members/Internal Health Care Providers	Elders/Knowledge Carriers	External Health Care Providers	Total
FWFN	15	17	15	9	
NFN	7	20	10	8	
PFN	26	25	25	10	
SNGRT	14	20	18	8	
Total	62	82	68	35	185

*FWFN* Fort William First Nation, *NFN* Naotkamegwanning First Nation, *PFN* Peguis First Nation, *SNGRT* Six Nations of the Grand River Territory

#### Community-specific analysis

The data were first analyzed separately for each community to provide communities an individual report of their findings. One member of the research team (HP) analyzed all quantitative data. Two members of the research team (HP and MLK) reviewed the qualitative data (focus groups and interviews) and did all analysis. This ensured consistency in the analysis across the four communities.

Analysis of the survey data included the calculation of descriptive statistics on the numerical data obtained from the closed-ended survey questions and thematic analysis of the qualitative data from the open-ended survey questions. The focus group and interview data were thematically analyzed line-by-line to identify ideas and themes specific to each community. A three-part inductive analysis process was used which included line-by-line analysis of the transcript to identify the ideas represented during the focus groups and interviews, grouping the ideas into themes and subthemes, and organizing the themes to address the research questions. [58, 59]. Member checking was used to validate the themes, and ensure the voice of the community was accurately interpreted [60]. Quotes selected to illustrate themes were edited to remove filler words (e.g., like, and, ah, um) to improve clarity without changing the meaning. A report was prepared for each community profiling community specific findings and recommendations. All final analyses and reports were approved by the community advisory committee prior to release to ensure accuracy.

#### Comparative community analysis

A comparative analysis was undertaken on the individual needs assessments. The findings in the individual community reports were reviewed and a table was created to compare the themes in each report to identify common themes among the communities.

### Phase 2: developing recommendations based on the findings

The completed analyses of the community assessments were used to create recommendations for action to guide the development of the palliative care programs. Recommendations were organized according to the themes that emerged during analysis of findings. The community advisory committee reviewed preliminary drafts and worked with the researchers to develop the final recommendations.

### Phase 3: developing and implementing educational resources

Recommendations included developing an education plan to address the identified educational needs of community members, internal health care providers and external health care providers. Based on each communities’ education plan, multiple tools and resources were created collaboratively by the community advisory committee, the community research lead, and the researcher team. These tools and resources were implemented incrementally over four years. Implementation included ongoing process evaluation to assess if the education (content and format) was useful, understandable, culturally appropriate and practical to implement. Revisions were made as required. Resources were implemented again until they were judged acceptable for use by the community advisory committee. Once finalized, the resources were included in a Workbook that is available to guide palliative care development in other First Nations communities [51].

## Results

### Phase 1: educational needs

Similar themes emerged in both the surveys and focus groups/interviews across the four communities. The integrated findings are presented here. Where one community had unique findings, this is acknowledged. The individual community reports can be accessed on the website [2].

**Fig. 4:**
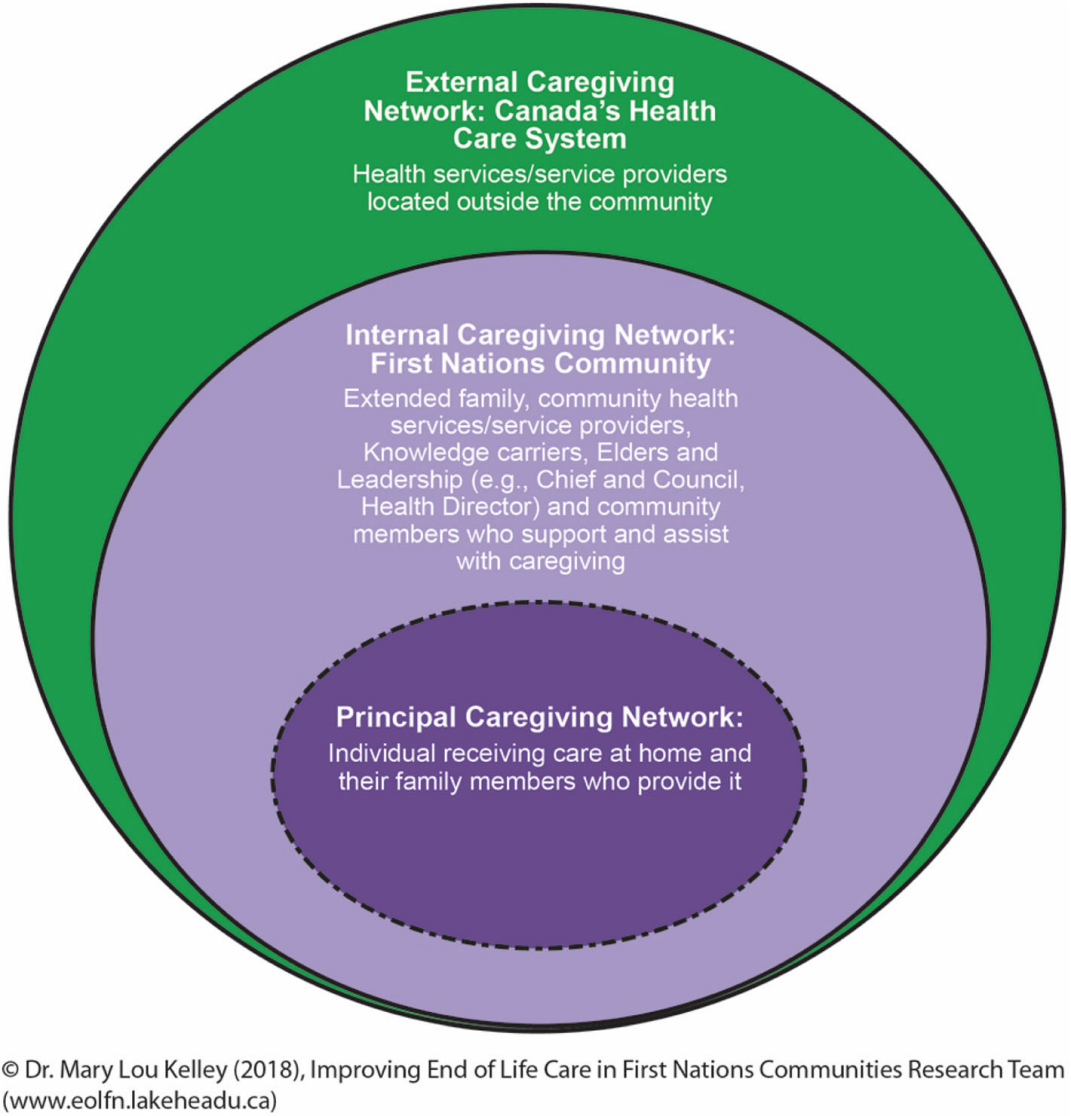
Palliative Caregiving in First Nations Communities. Note: The Palliative Caregiving in First Nations Communities model is an adaptation of Abel and colleagues Circle of Care Model depicting the health promoting palliative care approach [20]. Based on the EOLFN research, the Abel model was adapted for relevance to the First Nations community context [1]

Training and knowledge sharing was viewed as being a means to improving palliative care in all four First Nations communities. Several learnings emerged related to the education and training needs of community members and external health care providers. Those learnings are presented below using the Palliative Caregiving in First Nations Communities Framework (Fig. 4) to organize the findings.

#### Educational needs of clients and families (principal caregiving network)

Lack of knowledge was identified as a barrier to community members being able to die at home. Specifically, a lack of knowledge around services that are available in the community and a lack of knowledge regarding how to provide palliative and end-of-life care. Participants felt that community members diagnosed with a terminal illness and their families need to be educated on the options that are available to them. It was expressed that people also need to be educated on the possible outcomes of their decisions when choosing to remain in the community to receive care versus leaving the community for hospital or hospice.

“…I think the systems need to be in place so that family, when it comes to that time, know exactly what, what their direction would be. Cause right now I don’t think that that a lot of them know what’s available, whether that’s even an option. They just probably right now think, hospital”. (Community Member)

Participants expressed a need for education and resources for family members focused on how to support a dying family member. It was felt that being informed about their loved ones’ disease, what to expect, and how to provide care would assist in coping. Specific areas of focus identified were illness-specific information, personal care techniques (e.g., turning, lifting, bed sore treatment) and palliative and end-of-life care (e.g., pain medication and management in the home, using oxygen, and what to expect when a person is dying).

“I think a big part of it is talking to them, the families and, educating them ahead of time what to expect as well. Because it can be scary, frightening for families especially when we see them dying you know, or in pain or struggling. Just providing that knowledge and support ahead of time instead of in the moment when they’re stressed or anxious about what’s going on. Just preparing them gradually before it gets to that point … is very important.” (Internal Health Care Provider)

“I got this thing in my mind about a [palliative care] booklet. To me a booklet is a reference for yourself that you may need when you feel you need it, because the access to information is not always there to go and get. When you have a question come up or process, especially as things progress more intensely. But right at the beginning, I am a believer, if you understand, you cope better. So, if you understand the whole process, you are going to cope better with it and you will recognize and identify your needs better. Whether you are the caregiver or whether you are the person who has got the terminal illness and if it’s accessible to the whole family…” (Community Member)

Participants also felt that families need to know what services are available in the community for families and for those caring for a loved one. They also wanted to be better informed of upcoming training and education opportunities being offered within the community.

#### Educational needs of the First Nations community (internal caregiving network)

Lack of knowledge and education of internal health care providers and the community as a whole was also identified as a community level barrier to dying at home. Participants identified educational needs related to the community as a whole, internal health care providers, and community leadership.

##### Community members

Participants identified several educational needs that, if addressed, would benefit the whole community. It was expressed that traditional teaching and greater awareness of cultural knowledge could support community members in dealing with death dying and grief. Further, cultural knowledge would assist in alleviating some of the fear associated with death and practices around death. One of the impacts of colonization was disruption of the traditional intergenerational passing down of cultural knowledge that supports community members in dealing with death, dying, and grief.

“I think there’s things in our culture that are meant to help us, and we just have to be able to accept them. So that’s the learning that I want to take place…cause a lot of its fear. There’s a lot of fear out there, and it doesn’t have to be that way.” (Community Member)

The lack of communication about death and dying in one of the communities was perceived as a barrier. Participants suggested that community education is needed to address this issue. It was believed that being more open to talking about death and dying would lead to better care, and a greater ability for community members to stay at home when they are ill.

“it’s a difficult topic for anyone … across all cultures… Is it a stigma that we kind of put on it, just … not to talk about it, or is it [an] actual, cultural thing where … its part of the culture just not to talk about it? I think we need to address those kind of barriers, and see where everybody stands, sometimes individually even. I think just educating our community as a whole too in assuming we got issues like that. The easier it is, the more successful that person will be so that they can stay at home, because issues that are difficult to talk about only get more difficult when someone’s ill”. (Community Member)

Participants expressed that all community members need information and knowledge about what services are available to them, both in in the community and outside of the community. They also identified the need to educate community members on various topics related to palliative care (e.g., what to expect at end-of-life, roles and responsibilities of different health care providers, grief and loss, advanced care planning, power of attorney, and wills). A variety of formats for the needed education were identified (workshops, booklets and pamphlets, sharing circles, etc.). Participants also felt there is a need to educate children and youth around death, dying and end-of-life care.

##### Internal health care providers

Participants believed that all health care professionals – including doctors, nurse practitioners, nurses, and support workers-- could benefit from palliative care training, including training on providing compassionate care and on when to initiate the palliative approach to care. Participants explained that there was a lack of trained staff in the communities. It was expressed that more people in the community should be trained in health service roles generally, and palliative care specifically, including having palliative care training offered to volunteers. Internal health care providers viewed training as an important way to make improvements in care and to increase safety and quality of care for clients. Training needs identified included the technical aspects of care provision (e.g., on the use of pain pump technology) as well as the compassionate aspects. The following quotes illustrates the need for training to move beyond the technical and the importance of training in compassionate care.

“I think when you’re dealing with palliative care, I think it’s so important that caregivers, health care aides, nursing, get the proper training on compassionate care … for that individual. It’s not a job, it’s giving that care, the best quality care, end-of-life care. It’s so important, you know.” (Internal Health Care Provider)

“You can give ‘em any kind of medical instructions that they need. But if they don’t know how to care, feeling wise, loving wise, if you want … they have to invest themselves, because that’s a big investment, when somebody comes in, and they don’t care, it’s just, robotic. And so they do the functional things that they do, but, and I do believe this that everybody, who’s going through the end-of-life experiences know, and that’s a lie to them, when you only do the functional things, they know, they know.” (Knowledge Carrier)

Some internal health care providers expressed the desire to receive training in communicating about psychosocial issues related to death and dying with both clients and their family members.

“I was thinking too, about dealing with the loneliness sometimes and the depression and the psychological issues. Some of us aren’t trained to deal with those issues with the clients, so that would be an area of improvement for staff.” (Internal Health Care Provider)

Additionally, internal health care providers in one of the communities expressed a desire to be more knowledgeable about the different community beliefs and rituals, such as Longhouse, to be able to better serve their patients and family members.

##### Band leadership (Chief and council members, health directors and band managers who supervise staff)

Participants from one community identified the need to educate community leadership so that they could better support palliative care development in the community. They also felt there needs to be sensitivity training and increased understanding by Band Council and upper management about the grief that accompanies providing palliative care for the front-line staff during in their work. (It is noteworthy that many health care providers in First Nations communities are caring for their relatives at the end of life.) For example, one participant stated:

“For upper management and band councils, not just our band council, but band councils in general, … they lose that focus, they lose that sensitivity, that compassion. They say we’re a caring community. Again, I tend to disagree. When things, issues like this, come up and you have a hard time just saying ‘I need to be away, I need that time off’ and even after you get your week, you still don’t … end your grieving at five days …” (Community Member)

#### Education needs of Canada’s health care system (external caregiving network)

The external caregiving network refers to the health care system outside of the community. This includes physician services, hospitals, long-term care and other specialized services that are provided primarily by non-Indigenous providers (Fig. 2). The community assessments identified that First Nations people experienced a lack of culturally safe care. Cultural safety refers to an environment that is safe for people: there is no assault, challenge, or denial of a person’s Indigenous identity, who they are or what they need [61]. Participants also viewed their health care providers as lacking cultural competency when being cared for in the external network. Cultural competency refers to person’s ability to understand, appreciate, and interact respectfully with Indigenous peoples and their cultural teachings and traditions [51].

A lack of culturally safe and competent care was perceived as health system barriers to First Nations people wishing to be discharged to die at home. Participants expressed a need for more experiential education and training on providing culturally appropriate and safe care to First Nations people outside of the community (e.g., in hospitals in urban settings). It was felt that external health care providers need to learn about First Nations cultures and respect all cultural practices into their programs and policies.

“We need that linkage, we need stronger linkages with the doctors and the staff, the people off-reserve and on-reserve, so they can understand you know. They always talk about the communities but they’ve never been here, they’ve never experienced it, so, how do they know?” (Community Member)

“A lot of the places don’t allow our large families to come and sit or stay or be there. They, as soon as they seem them coming, they’re like, we’re only letting one person in at a time, that’s the downfall of going somewhere else.” (Community Member)

### Phase 2: recommendations for palliative care education

Based on the participants’ perceived needs, a series of recommendations were formulated by the researchers and advisory committees to advance palliative care program development, and expand the palliative care program in each community. These included specific recommendations that could guide future actions related to developing palliative care practice, policy, and education (See Table 5).

**Table 5:** Summary of Education Related Recommendations to Advance Palliative Care Program Development in the Communities

Overarching Recommendation*:* The palliative care program needs to be advanced and supported by creating a culturally appropriate palliative care education and training plan along with educational resources to implement it. The plan needs to include providing annual education for the: Principal and Internal Caregiving Networks (family caregivers, community members, including internal health care provides), and the External Caregiving Network (external health care providers who service the residents of the First Nations community).	
The education plan for the Internal and Principal Caregiving Networks should include, but is not limited to:	- Understanding palliative and end-of-life care as a continuum of care that begins when someone is identified as having a life-limiting illness. - Knowledge and skills for providing palliative and end-of-life care. - Culturally appropriate palliative care practices for community residents. - Training on advanced care planning and how to have advanced care planning discussions with individuals and families. - Illness specific information (e.g., disease progression, illness specific resources). - Education about grief and bereavement support strategies. - Family education and support related to caregiving, advance care planning, preventing caregiver stress, and managing grief and loss. - Community education on topics such as wound care, what to expect at end-of-life, roles and responsibilities of different health care providers, grief and health education in general.
The education plan for External Caregiving Network should include, but is not limited to:	- Understanding the historical context of living in a First Nations community, the structure and organization of First Nations communities in general and health services in particular, federal funding of health service delivery, and funding of medical equipment. - Understanding the importance of kinship/relationships and taking time in their care of the First Nations community clients and families. - Developing skills in culturally effective communication strategies to ensure that clients, families, and community members fully understand and are aware of decisions and health care services. - Understanding that for some First Nations clients, talking about death and dying is not culturally acceptable and knowledge of alternative strategies when providing services. - Understanding that First Nations clients may have different understandings of health and illness. - Understanding that the spiritual and cultural practices of individuals and families are highly individualized and health care providers should proactively ask what practices would offer comfort and support. - Understanding what services are available within First Nations communities and how to refer clients to these services. - Understanding that provincial health services have an obligation to provide palliative care services to residents living in First Nations communities.

### Phase 3: developing and implementing educational resources

Multiple community-led initiatives were developed and implemented to address the educational needs and recommendations stemming from phases 1 and 2. Those initiatives (mapped against the educational needs identified in Phase 1) are presented in Table 6. All educational resources can be found on the EOLFN website [2].

**Table 6:** Summary of Community-led Initiatives Developed to Address the Educational Needs Identified in the Community Assessments

Identified Educational/Training Needs	EOLFN Education Strategies/Resources Created^a^
Principal Caregiving Network	
Clients and families knowing options and available resources for receiving care (hospital vs home vs hospice) to make realistic choices. Knowing what services and supports are available in the community to support dying at home.	Each community created a brochure for clients and families describing the local palliative care program (eligibility criteria, care pathway and services offered) and palliative care experts/resources external to the community that could be accessed. This brochure and an information poster were distributed throughout the community by the Community Lead and project advisory committee.
Getting palliative care specific information on caring for people with a terminal illness/ expected death at end of life and how to prepare.	Distributed a set of culturally appropriate palliative care brochures on the following topics: what is palliative care; living with a terminal illness; caring for someone with a terminal illness: care for the caregiver; caring for someone with a terminal illness: what to expect; supporting the caregiver and the family. Developed and distributed (in collaboration with the Canadian Hospice Palliative Care Association, the Way Forward and the Quality End-of-Life Care Coalition) a series of culturally appropriate educational resources on Advance Care Planning. This included a poster, a culturally appropriate workshop with slides and facilitator guide, an educational video, and set of 2 brochures on advanced care planning and substitute decision-making.
Internal Caregiving Network: First Nations Community	
Knowing community cultural practices and traditional teachings related to death, dying, loss and grief. Knowing how to talk about death and dying in a culturally appropriate way, including internal health care providers heath care providers.	Engaged Elders and Knowledge Carriers to share cultural knowledge and model culturally appropriate conversations about death and dying at workshops, community meetings/events. Opening and closing prayers or smudging were part of each event. Creating culturally specific print and video resources about cultural traditions and practices relating to death, dying, loss and grief.
Knowing enough about palliative care so there is not fear of death and dying. The community understanding what palliative care involves. Knowing what palliative care resources are available. Band Leadership learning about palliative care and how to better support local palliative care development and the staff, families and community caregivers.	Members of the local palliative care team/program in the community were supported and mentored by regional palliative care consultants to provide bedside education about providing palliative care: physical care, practical care, end-of-life care/death management, loss/grief, spiritual care, social, psychological. Developed and distributed culturally appropriate palliative care booklets and pamphlets throughout community at health fairs, public meetings. They were developed in collaboration with First Nations community members and are in plain language for the general public. Initiated palliative care program logo contest to engage community members and promote awareness of palliative care. Community meetings held to launch palliative care development initiative. Providing a series of free community awareness sessions facilitated by internal and external health care providers and palliative care experts. Topics included: introduction to palliative care; supporting the family; managing pain and symptoms; care for the caregiver, providing care at home. Engaged Band Leadership to participate in community meetings/committees about palliative care and attend community awareness sessions. Band Leadership learn about palliative care, how to better support local palliative care program development and how to support the staff, families and community caregivers who provide palliative care (e.g. staff experience grief and may need additional time off).
Existing community health care providers become better trained in skills for providing palliative care, communication, counselling and psychological care to clients and families (e.g. loneliness and depression) especially related to death and dying.	Regional palliative care experts taught First Nations health care providers how to use practice tools (e.g. assessment forms, protocols for dying at home) and implement care practices commonly used in palliative care (e.g. client care conferencing, care planning). These palliative care experts volunteered to provide education during the needs assessment phase of the project, and they coached/mentored the home care staff in the First Nations communities (The regional palliative care experts were employed in hospices, cancer centres, hospitals and home care agencies). Provided culturally appropriate educational course *Palliative Care for Front Line Workers in First Nations Communities.* Topics included: creating context, working with families, pain and symptom management, when the time is near, grief and bereavement, helping relationships, community care teams. A workshop *Finding our Way Through: Navigating Loss and Grief in First Nations Life* was created by a member of the research team and was offered to heath care providers in First Nations communities. This experiential workshop involves participants in developing a circle of trust and feeling a sense of safety and trust in a group of people. The workshop requires a skilled facilitator and the involvement of Elders and Knowledge Carriers, and is designed for adults age 18 and over.
External Caregiving Network: Canada’s Health Care System	
All external heath care providers need more training about: the cultural practices of the client population they service, implementing culturally safe [61] practices, and offering culturally appropriate compassionate care. Data emphasized the need for better communication and social support/care.	External heath care providers who provided care in the community were invited to participate in a journey mapping event to create the palliative care pathway for home care. Evaluations indicated that through journey mapping they learned how the community provides care in the First Nation, what local health service available, and how to integrate their services in a culturally appropriate way [43]. External heath care providers were invited to participate in full day cultural awareness workshops hosted by the First Nations community. Local Elders and Knowledge Carriers and local heath care providers shared cultural practices and expectations with external heath care providers in a sharing circle format with story telling and answering questions posed by external heath care providers. External heath care providers were invited to participate in care conferences with the client, families and First Nations heath care providers, allowing external heath care providers to better understand how to integrate their expertise and services in a culturally appropriate way.

^a^Examples of the initiatives can be found on the project website [2]

## Discussion

The World Health Organization advocates that all people deserve to have the best possible quality of life at the end of life, including people living in Canada’s First Nations communities [62]. The public health approach to palliative care (health promoting palliative care) strives to accomplish that by viewing palliative care through a social and cultural lens and shifting the responsibility for the provision of palliative care from institutions to communities. Implementing the public health approach requires normalizing death. Normalizing death requires educating families, community members, and generalist health care providers and providing them the resources they need to care for and support dying community members and their families. This paper presented community-based PAR in four First Nations communities in Canada that assessed and addressed local palliative care educational needs to improve community capacity in palliative care. This research contributes to the field by illustrating the public health approach in the Canadian, First Nations context, and offers implications for practice, policy, and research. Those implications are discussed next.

### Practice and policy implications

Despite a growing need, First Nations people in Canada face many barriers to accessing quality palliative care. In their recent review of the international literature, Caxaj et al. concluded that building local capacity to provide more relevant and culturally appropriate palliative care is a key priority for improving palliative care for people living in Indigenous communities [63]. The research presented addresses that priority by providing an example and evidence to support the benefits of a community-focused, public health approach that is implemented in communities, from the bottom up and inside out. It illustrates a process that can be used by health care decision makers and policy makers to improve access to quality palliative care in First Nations communities.

The community assessments identified educational needs and based on these needs, culturally appropriate educational resources were developed and implemented to build capacity to care for and support community members through death, dying, loss and grief (Table 6). The communities have shared all their resources in the *Developing Palliative Care Programs in First Nations Communities: A Workbook,* available at no cost on the EOLFN project website [2]. Palliative care is a medical concept unfamiliar to First Nations people because traditionally First Nations people do not medicalize death [35, 37, 38, 40]. The resources were all culturally appropriate because they were developed through PAR in First Nations communities by First Nations people, for use by First Nations people. The Workbook and educational resources are presented in a very accessible and useable toolkit format for use by all communities that wish to build their local capacity to provide culturally appropriate, community-based palliative care.

Health promoting palliative care promotes improving communities’ death literacy, i.e. the knowledge and skills necessary for understanding end of life issues, including what services exist and how to use them [64, 65]. This research contributes culturally appropriate strategies for improving the death literacy of First Nations’ communities. For example, advanced care planning is a concept in medicine that does not fit well with some First Nations cultures  as some communities' traditional teachings do not include planning for death. Families have reported the process of advance care planning is “like an imposition of an individualist framework on what should be a family decision” [63]. Through this research, focus groups, community consultations and evaluations guided the creation of culturally relevant advance care planning resources. Video and print resources focus on choosing a substitute decision maker (spokesperson or proxy) as opposed to planning end-of-life care. Simple, non-legal language is used, and emphasis placed on the benefits to the family of knowing their loved one’s wishes. Our educational resources not only support First Nations people to provide palliative care in their community, but also prepare them if they engage with the Western, medical system of palliative care.

Our research also provides a specific example of improving death literacy through the combination of community development (capacity building) and education. Improving community death literacy is attained through a combination of experiential and educational learnings, and mobilizing and developing existing community knowledge and capacity [64] as described in this research. Once developed, death literacy becomes a resource for individuals and communities to support dying people and their families through the death system [64]. The culturally relevant and culturally appropriate resources developed and implemented through this research improve community capacity by building First Nations peoples’ death literacy.

Another implication of this research is for the training needs of external health care providers. Previous research has identified differences between Western (individualistic) and Indigenous (collective) worldviews as a challenge of providing Indigenous palliative care [15, 64]. This research further highlights the need for external health care provider education to facilitate culturally safe [61] care for First Nations people and provides examples of how to engage and educate external non-Indigenous health care providers.

Given the diversity of First Nations values and beliefs and the lack of alignment between the values implicit in Western medicine and Indigenous people [15, 64], we endorse a relational approach which focuses on cultural humility as opposed to cultural competence [66]. Cultural humility is a process of self-reflection and discovery in order to build honest and trustworthy relationships [67]. It calls upon the non-Indigenous health care provider to avoid a stance of superiority towards an individual’s cultural background and experience [68] in favour of adopting a stance that acknowledges and respects the potential differences in the meaning of health and wellness among people [69]. We endorse the recommendations for non-Indigenous health care providers made by Johnston et al.: 1. Learn the history of Indigenous people and its relevance to health care today (impact of colonization; gaps and issues in provision of health services); 2) Gain insight into the views of the Indigenous person (make an effort to get to know the person, treat each situation as unique and case-specific, recognize the value placed on trust and respect, be aware of non-verbal communication and cues for discussion, consider the role of religious beliefs and spirituality [15].

Our findings emphasize that dying is a significant cultural and spiritual life transition for First Nations people and end-of-life care is appropriately viewed through a social lens. The need for excellent pain and symptom management must be addressed by health care professionals who are respectful of their world view and will support efforts to provide end of life care in the home wherever possible and desired. Care in the home overcomes many of the institutional and cultural barriers for First Nations people identified by Caxaj [63], and also maintains important family connections.

The recommendations that were developed as part of this research (Phase 2) have implications for health care policy and health care decision makers at the local, provincial and federal government levels. Resources and support are required from all three levels of government to implement the initiatives described in the Workbook. Based on our research, our guiding policy principle is to integrate the knowledge and best practices of two systems using capacity development strategies, namely the First Nations communities (e.g., their vision for palliative care; Indigenous understandings of death and dying, traditional caregiving practices) and the Western medicine system (e.g. their palliative care training and education, pain and symptom management, specialized palliative home care teams and programs). This policy principle was inspired by the concept of Two-Eyed Seeing articulated by Mi’kmaw Elders Albert and Murdena Marshall. Implementing this principle, one eye sees using Indigenous ways of knowing, and the other sees using Western perspectives. The principle of Two-Eyed Seeing is based on a “dynamic, changing, interaction and relational process which generates new ideas, understandings and information” [70, 71]. This idea is further described in another publication [1].

### Research implications

Community capacity building and education are key strategies for implementing the public health approach. This research contributes an example of those strategies. According to the public health approach, palliative care education needs to be developed in partnership with the community [64], as described in this research. Thus, this research contributes a methodology for assessing and addressing local palliative care educational needs in partnership with communities. The community assessments were community controlled and culturally appropriate. Though there was consistency amongst the educational needs, the communities took different approaches to addressing those needs. A key element of the approach was the partnership *with* the communities -- the communities led the assessments and the development and implementation of the educational initiatives.

This research also illustrates the benefits of PAR as a methodology to developing local palliative care capacity. PAR aims to create social change; it is particularly valuable for facilitating community capacity development as the research is embedded in social action. In PAR the researchers and community members work together to co-create locally relevant knowledge through relevant reflective spiral of activity; policy and practice change occur as part of the research process [1, 56].

This research also illustrates the value of the Kelley model for community-based palliative care capacity building [57]. The model provides a theory of change that can be used to guide the palliative care development process in other communities. Education is a key component of the broader community capacity development approach. That education needs to be grounded in a culturally appropriate understanding of the community understanding, assets, resources and needs. A unique aspect of the approach described in this research was to develop a wide and comprehensive range of educational resources. Topics included all aspects of palliative care – physical, psychosocial, spiritual, and navigating the health care system. The education targeted all aspects of the caregiving network – the patient, family, community members, and internal and external caregivers (Fig. 4). Consistent messaging was delivered throughout to facilitate shared understanding and community mobilization.

This paper focused on the role of education in implementing the public health approach to palliative care in First Nations communities. Specifically, the focus was on the educational components of the community assessments, and on the community-led initiatives (tools/resources) that were developed to address the identified educational needs. We would like to emphasize that though education is essential, education alone is not enough. Successful implementation of the public health approach also requires education of policy makers, health care providers and the community as well as appropriate policies and integration of palliative care services throughout all levels of society [22]. Each element of the public health approach was implemented in the EOLFN research, as described in a separate paper which provides an overview of all components of the EOLFN research [1].

## Conclusion

In Canada, there is a growing need to develop community-based, culturally appropriate palliative care for Indigenous people living in First Nations communities. With an emphasis on social and community care, the public health approach to palliative care is relevant. The research presented here offers an example of the public health approach in four First Nations communities in Canada, and offers implementation strategies around palliative care education (a key strategy in the public health approach). The emphasis was on community capacity building through education; the focus was on identifying needs and presenting solutions. Implementation strategies are offered around assessing educational needs and addressing them. Our hope is that the community assessment methodology, case study of educational needs, and the examples of resources and strategies for addressing those needs presented here will be helpful for those looking to develop palliative care programs and capacity in Indigenous communities in Canada and internationally.

## Additional file

Additional file 1: The surveys and focus group/interview guides that were used in the community assessments are included as Additional file. (PDF 224 kb)

## Abbreviations

CIHR: Canadian Institutes of Health Research
EOLFN: Improving End-of-Life-Care in First Nations Communities [research project]
FWFN: Fort William First Nation
NFN: Naotkamegwanning First Nation
OCAP: Ownership, Control, Access, Possession
PAR: Participatory Action Research
PC: Palliative Care
PFN: Peguis First Nation
SNGRT: Six Nations of the Grand River Territory
