# Supplementary material for: “If you understand you cope better with it”: the role of education in building palliative care capacity in four First Nations communities in Canada
Source: BMC Public Health. 2019 Jun 17;19:768. doi: 10.1186/s12889-019-6983-y (PMC6580639; doi:10.1186/s12889-019-6983-y)
Supplement: Supplementary file 1 — The surveys and focus group/interview guides that were used in the community assessments are included as Additional file. (PDF 224 kb) [file 12889_2019_6983_MOESM1_ESM.pdf]

Additional file 1

EOLFN Community Survey & Interview/Focus Group Guides

1. COMMUNITY ASSESSMENT SURVEY*

1. Gender:
- ☐ Male
- ☐ Female

2. Age:
- ☐ 18 – 30
- ☐ 31 – 40
- ☐ 41 – 50
- ☐ 51 – 60
- ☐ 61 and over

Your General Knowledge of Palliative Care

3. Have you heard of the term Palliative Care?
- ☐ Yes
- ☐ No

For the purposes of this survey, the term "palliative care" is defined as the following:
Palliative Care: **A term used to describe a variety of services that are brought together to relieve the suffering and improve the quality of life for persons living with or dying from a terminal illness. These services are also available for family members of the individuals.**

4. Who do you think that palliative care services are intended for? (please choose one answer)
- ☐ Everyone at the end of their life regardless of their illness
- ☐ Only patients dying of a life threatening disease like cancer or AIDS
- ☐ I don't know

5. Where do you think that palliative care is offered? (please check all that apply)
- ☐ Long-term facility or nursing home
- ☐ Hospital
- ☐ Patient's home
- ☐ Hotel
- ☐ In the Community
- ☐ Other ____

6. What services do you think palliative care includes? (please check all that apply)
- ☐ Medical services such as pain management
- ☐ Psychological support such as dealing with depression and anxiety
- ☐ Home-making such as help with household tasks, cooking, cleaning, etc.

- ☐ Spiritual care such as dealing with beliefs/religious practices/traditional customs
- ☐ Personal care such as hairdressing/shaving
- ☐ Respite
- ☐ Other ______________________________

Your Personal Opinions

7. If you needed information about services for someone who is dying, who would you go to for information? (please check all that apply)

- ☐ Family Doctor
- ☐ Community Health Centre
- ☐ Health Nurse
- ☐ Hospital
- ☐ Pharmacist
- ☐ Internet
- ☐ Tribal Authority Health Clinic
- ☐ Community Health Representative
- ☐ Friend/Family Member
- ☐ Social Worker/Counsellor
- ☐ Priest/Minister/Pastor
- ☐ Traditional Healer/Elder
- ☐ Other ____________

8. Have you or a family member ever provided care for someone who was dying? (*if No, please go to question #9*)

☐ Yes ☐ No ☐ I don't know

a) Where was this care provided at? (please check all that apply)

- ☐ Long term care facility or nursing home
- ☐ Hospital
- ☐ Home
- ☐ Other ______________________

b) Did you use health or community care services to take care of the person who was dying?

☐ Yes ☐ No ☐ I don't know

If yes, please name the services that you used ______________________

____

____

____

c) How satisfied were you with the services that you and your loved one received? (please circle one answer)

Very Satisfied Satisfied Dissatisfied Very Dissatisfied Don't Know

9. How many hours a week do you think it would take to care for a dying loved one in your home – this would include tasks such as food preparation, housekeeping, shopping and attending appointments?

______________ hours a week

10. Do you think you could devote this much time to caring for a dying loved one, given your current schedule?

☐ Yes ☐ No

If no, please indicate why not? ____
____
____
____
____

11. Do you feel that talking about death and dying is acceptable in your community?

☐ Yes ☐ No ☐ I don't know

If no, please indicate why not? ____
____
____
____
____

12. In your opinion, how important is it to discuss your end-of-life care with a family member? (please circle one answer)

Very Important	Important	Not very important	Not at all important	Don't Know

13. Have you ever discussed your end-of-life care with a family member?

☐ Yes ☐ No

14. In your opinion, how important is it to discuss your end-of-life care with a doctor? (please circle one answer)

Very Important	Important	Not very important	Not at all important	Don't Know

15. Have you ever discussed your end-of-life care with a doctor?

☐ Yes ☐ No

16. In your opinion, if services were available and adequate, would more community members choose to die at home?

☐ Yes
☐ No
☐ I don't know

17. Which comes closer to the way you feel: (please choose one answer)

☐ People should start planning for end-of-life care when they are healthy
☐ People should start planning for end-of-life care when they have a serious illness
☐ You can't plan for end-of-life care; planning happens when you need it

18. What would be some of the advantages of choosing to die in your community?

____
____
____

19. In your opinion, what additional services or programs are needed to improve the experience and care of people who are dying in your community?

____
____
____
____

20. What kinds of education or training do community members need to support people who choose to die at home in your community?

____
____
____
____

21. If you needed to get services in the home for someone who is dying, where would you go or who would you talk to?

____
____
____
____

22. Any Additional Comments:

____
____
____
____

** Some questions in this survey were adapted and revised from a Ipsos-Reid survey conducted in 2004.*

The Glaxo-SmithKline Foundation and the Canadian Hospice Palliative Care Association. (2004). Ipsos-Reid Survey. Hospice palliative care study: Final report, January 2004.

2. COMMUNITY ASSESSMENT INTERVIEW / FOCUS GROUP GUIDES

2.1. *Guide for community members*

1. Within your community, what is the primary diagnosis of terminally ill people?
2. Where do you feel that community members would prefer to die?
3. What would be some of the advantages of dying in this community?
4. What do you feel is the role of the community and family members when someone is dying?
5. What would be some of the challenges/obstacles that would prevent people from choosing to die in this community? What does your community need to allow terminally ill people to return home to die?
6. In your opinion, what can be done to improve the experience and care of people who are dying in your community?
7. Do you think that talking about death and dying is acceptable in your community?
8. What are some of the beliefs and values surrounding death and dying in this community? What are some of practices that are customary when someone is about to die?
9. What are some of your experiences working/providing care in your community for care with people who are dying?
10. Follow-up questions to the survey exploring contradictions will also occur. This will include topics such as: The surveys indicate that people in your community feel that discussing end-of-life care wishes (with family or your doctor) is a very important thing to do but also that it is not being done very often.
    i. Can you explain this contradiction?
    ii. Why are these discussions not taking place?
11. Is there anything else that you would like to add on the topic of palliative care in your community?

2.2. Guide for Elders and Knowledge Carriers

1. How can I use this information that you are going to tell me?
2. From your perspective, what is the meaning of health and illness?
3. Is talking about death and dying acceptable in your community?
4. As an outsider coming in to your community, what do I need to know if I were helping or providing care for someone who was dying in your community?
5. What do you feel is the role of the community and family members when someone is dying?
6. What are your beliefs relating to advance health care planning?
7. What does your community need to know or take into consideration when developing a palliative care program?

2.3. Guide for First Nations (internal) health care providers

1. Why does your community need palliative care? What events have led your community to want develop a palliative care program?
2. What services do you and/or your organization currently provide in the First Nation community?

3. How are these services accessed by patients and families? What are the eligibility requirements?
4. What organizations are you currently partnered with to provide service?
    - What is the decision making structure with your partners?
    - When and how did these relationships develop?
    - Are there formal service agreements and/or MOU's with these agencies?
    - What resources can be accessed through your partners?
5. Does your community receive palliative care services? If so, from where?
    - Does your community have its own palliative care program?
    - Who provides funding for palliative care services? Does the First Nation band directly receive the funding? If so, from where?
    - Are external agencies funded to provide palliative care services?
    - Are there service overlaps/gaps?
6. What do your health staff know about palliative care? Have they received any special training? If so what, when, and from where?
7. Who has received palliative care training in the community?
8. What do you see as the barriers and opportunities to providing enhanced palliative care and developing a palliative care program in this First Nation community?

2.4. Guide for external health care providers

1. What services do you and/or your organization currently provide in the First Nation community?
2. How many residents of the (Name of First Nation community) access services from you and/or your organization? (if known)
3. How are these services accessed by patients and families? (may be in a variety of ways; Find out about all the different ways.)
4. What do you see as the barriers and opportunities to providing enhanced palliative care and developing a palliative care program in this First Nation community?
5. What is your and/or your organization's potential contribution to providing palliative care and developing a palliative care program for people who want to die in (name of First Nation community)?
